# Ecologic Factors Contributing to West Nile Virus Hyperendemicity in Central South Carolina: An Integrated Vector–Human–Environmental Study

**DOI:** 10.4269/ajtmh.25-0305

**Published:** 2026-02-05

**Authors:** Elba S. Fridriksson, Ahalya Muraleedharan, Kyndall C. Dye-Braumuller, Madeleine M. Meyer, Kia Zellars, Huixuan Li, Melissa S. Nolan

**Affiliations:** ^1^ Arnold School of Public Health, University of South Carolina, Columbia, South Carolina;; ^2^ Institute for Infectious Disease Translational Research, University of South Carolina, Columbia, South Carolina

## Abstract

West Nile virus (WNV) is an endemic arboviral infection in the United States that has undergone phylogenetic evolution since its introduction 25 years ago. An integrated vector–human–pathogen study was conducted in the summer of 2023 to unearth contemporary *Culex quinquefasciatus* habitat patterns and human transmission spillover foci in South Carolina, a state with historically little WNV data. A serosurvey revealed WNV seroprevalence 10 times the national average (22% versus 2%, respectively), with unusual epidemiologic risk factors. Female *Culex quinquefasciatus* WNV positivity was low (2.7%), with viral phylogenetics 100% homologous to the WN02 clade. Mosquito vectors clustered in affluent urban neighborhoods with greater tree canopy cover and abundant waterbodies. *Culex quinquefasciatus* abundance was greatest when climate variance was nominal in the 72 hours preceding collection. An unusual bimodal mosquito temporal pattern was observed, reflecting changing climate patterns. The present comprehensive WNV study reveals emerging transmission factors as WNV continues to evolve and persist in the southeastern United States.

## INTRODUCTION

West Nile virus (WNV), a flavivirus in the Japanese encephalitis virus complex, was first isolated from a febrile patient in the West Nile region of Uganda nearly 90 years ago.^1^^,^^2^ Although most infected people remain asymptomatic, <1% of infected people will develop neurological disease.^3^^,^^4^ West Nile neurologic disease (WNND) is characterized by acute meningitis, encephalitis, and acute flaccid paralysis. Among WNND survivors, most will retain permanent disabilities, including autonomic dysfunction, chronic kidney failure, depression, retinopathy, and chronic fatigue syndrome.^5^^–^^9^ Even febrile patients without diagnosed neurologic impairment are susceptible to long-term health impacts from the disease.^5^ Despite promising pharmaceutical trials in the early 21st century, a competent vaccine and treatment remain elusive.^10^

After its establishment in the United States in the early 2000s, this mosquito-borne viral infection has infected >7 million residents and killed ∼3,000 people in what were historically nonendemic counties.^11^^,^^12^ In the contiguous United States, three key *Culex* mosquito species serve as the prominent vectors, each associated with geographic locations and preferred avian amplifying hosts.^10^ The majority of US-based public health research has focused on the central states, ranging from North Dakota to Texas, where focal outbreaks have been routinely detected over the past quarter-century.^12^^,^^13^ Because of stabilization in annual case burdens and competing priorities for vector-borne diseases, WNV funding has declined considerably in the past decade.^14^ This decreased funding has subsequently led to a reduction in vector control efforts and likely exacerbated physicians’ already limited knowledge, culminating in an increased risk of undiagnosed disease transmission and outbreaks.^15^^,^^16^

South Carolina is a small- to moderate-sized state in the southeastern United States that is home to nearly 5.5 million people. West Nile virus was first identified locally in 2003, and mosquito, avian, human, and equine cases have been detected ever since.^12^^,^^17^ The scientific literature suggests that this state exhibits low-burden WNV endemicity,^11^^,^^12^ although it hosts rich ecological environmental pockets that support *Culex quinquefasciatus* (*Cx. quinquefasciatus*) mosquitoes and subsequent WNV transmission. To date, only five WNV clinical, veterinary, or epidemiology studies have been conducted in the state.^18^^–^^22^ Although brief, these studies have revealed the likelihood of sustained local transmission. After a fatal human case in Richland County in 2022,^23^ University of South Carolina investigators sought to close the scientific knowledge gap in local WNV transmission risk by conducting a comprehensive human–vector–pathogen study. The present ecological study was conducted in Richland County, SC, to evaluate potential microenvironmental factors contributing to incident and prevalent WNV human cases.

## MATERIALS AND METHODS

Environmental, mosquito, and human sampling efforts were incorporated in the present multipronged study (Supplemental Figure 1). This study was conducted in the summer of 2023 in Richland County, SC, with a geographic focus on the historically WNV-endemic downtown and the Columbia neighborhoods of Heathwood, Melrose Heights, Shandon, Hollywood-Rose Hill, and Rosewood. These neighborhoods were selected on the basis of expert commentary from the state public health entomologist and analysis of historical human case burdens. Residents of these five neighborhoods were invited to participate in any combination of the following activities: 1) a free WNV antibody test, 2) an exposure and health survey, and 3) free mosquito sampling from their domestic property. Residents were initially informed of the study’s activities through mailed postcards (sent in May 2023) that included a QR code for additional information. A list of resident addresses for the invitation postcards was purchased from MailersHaven, a third-party commercial list service used for a previous coronavirus disease 2019 study conducted in April 2023.

### Cross-sectional human WNV serosurvey.

Participants aged 18 years and older were invited to take part in a free dried blood spot-based WNV testing event. Participants were informed of the study through the aforementioned mailed postcard or through venue-based recruitment. Interested individuals who received the postcard were invited to either attend the in-person recruitment sites or contact the study headquarters and request an individual sampling appointment. In collaboration with community partners, in-person recruitment occurred throughout June 2023 at 1) a local craft brewery, 2) the local neighborhood library branch, and 3) a local grocery store serving racial minorities. Tents staffed with study personnel, equipped with educational materials and sampling kits for school-aged children and adults, were set up in visible, high-traffic areas outside these three establishments. After consent was obtained, a certified phlebotomist performed a capillary blood prick and collected four dried blood spots on a Whatman card. A self-reported health and exposure survey was also conducted, which included questions related to household mosquito and WNV-specific exposures, respondent WNV knowledge, mosquito abatement strategies used on one’s property, and household demographics and characteristics. Dried blood spots were temporarily stored at –80°C for 3 months. Samples were tested using Abbexa’s Human WNV IgG and IgM ELISA kits (Abbexa, Cambridge, United Kingdom). Seven participants did not provide enough blood material for both antibody assays, and their samples were prioritized for IgG testing only. Serologic testing was executed according to the manufacturer’s directions.

### Prospective WNV mosquito surveillance.

Local residents who received the mailed postcard were asked to indicate their willingness to permit mosquito surveillance on their domestic properties by scanning the postcard’s QR code, which linked to a REDCap site (Vanderbilt University, Nashville, TN). A list of all potential sampling locations was generated from information provided by postcard respondents from May 22 to June 12, 2023. A final selection of 25 homes was made by the investigative team to ensure environmental diversity and even representativeness among all five neighborhoods. Five homes from each of the five neighborhoods were sampled on a rotating basis from June 5 to September 25, 2023, for a total of 240 trap nights. Written permission was obtained from each resident before entering their property.

Weekly trapping efforts were performed as follows: 1) Reiter-Cummings gravid traps (Bioquip Inc., Rancho Dominguez, CA) with hay-infused water were set every Monday; 2) collection nets were collected the following day, and all live adult mosquitoes were stored in a freezer at –80°C until processing; 3) trap buckets were collected on Thursday, with any visible *Culex* mosquito egg rafts collected for laboratory rearing. On an Avantik EM^8^ Cryo-Console chill table (Avantik, Pine Brook, NJ), all adult mosquitoes were separated by sex, with females morphologically identified to species using a dichotomous key.^24^ Female *Cx. quinquefasciatus* mosquitoes were pooled in groups of ≤50 for WNV antigen detection using the commercially available rapid analyte measurement platform test (Response Biomedical Corp., Burnaby, British Columbia, Canada), according to the manufacturer’s protocol.

Any positive mosquito pools were subjected to next-generation amplicon sequencing using an Oxford Nanopore Technologies (Oxford, United Kingdom) Native Barcoding Kit on a MINION Mk1c device. A pan-flavivirus ∼252 base pair region of the *NS5* gene was amplified using the method described by Martinez et al.^25^ Consensus sequences were generated using Oxford Nanopore Technologies EPI2ME software and amplicon workflow with a WNV-02 reference sequence (GenBank Accession Number JF703161.1). Phylogenetic analysis was conducted using MEGA11 (MEGA Software, Dortmund, Germany).^26^ Sequences were aligned using MUSCLE (https://www.ebi.ac.uk/jdispatcher/msa/muscle), and the best evolutionary model for the study dataset was selected using MEGA11’s “Find Best DNA/Protein Models (ML)” analysis.^26^^,^^27^ A maximum-likelihood tree was constructed using the Tamura-Nei model with 200 bootstraps.^28^

### Statistical, geospatial, and remote sensing environmental analysis.

A comprehensive analysis was conducted to evaluate potential correlations among *Cx. quinquefasciatus* mosquito breeding habitat presence, *Cx. quinquefasciatus* mosquito abundance, and WNV human transmission clusters. Climate data, including humidity, wind speed, precipitation, and temperature, were generated for each mosquito collection date using the National Oceanic and Atmospheric Administration’s weather database. Environmental data, including the normalized difference water index, tree canopy coverage, surface temperature, and land use classification type, were collected through domestic surveys and remotely sensed variables within a 250 m buffer of the mosquito collection sites. Geographic weighted regression with Jarque-Bera tests and hotspot cluster analysis (Global Moran’s I and Getis-Ord Gi*) was performed to assess geospatial determinants of human seroprevalence, and Empirical Bayesian Kriging values for female *Culex* spp. mosquito counts were generated to compare human seropositive residents with mosquito abundance; these analyses were executed using ArcGIS Pro (ESRI Corporation, Redlands, CA). To evaluate positive WNV antibody results in relation to associated survey variables, logistic regression or analysis of variance was performed, depending on variable type. All variables with a *P*-value <0.25 in univariate regression were included in a backward stepwise multivariate model until only variables with *P*-values <0.05 remained; these analyses were performed using STATA v18.0 statistical software (StataCorp, College Station, TX).

## RESULTS

### Cross-sectional human WNV serosurvey.

**Figure 1.**
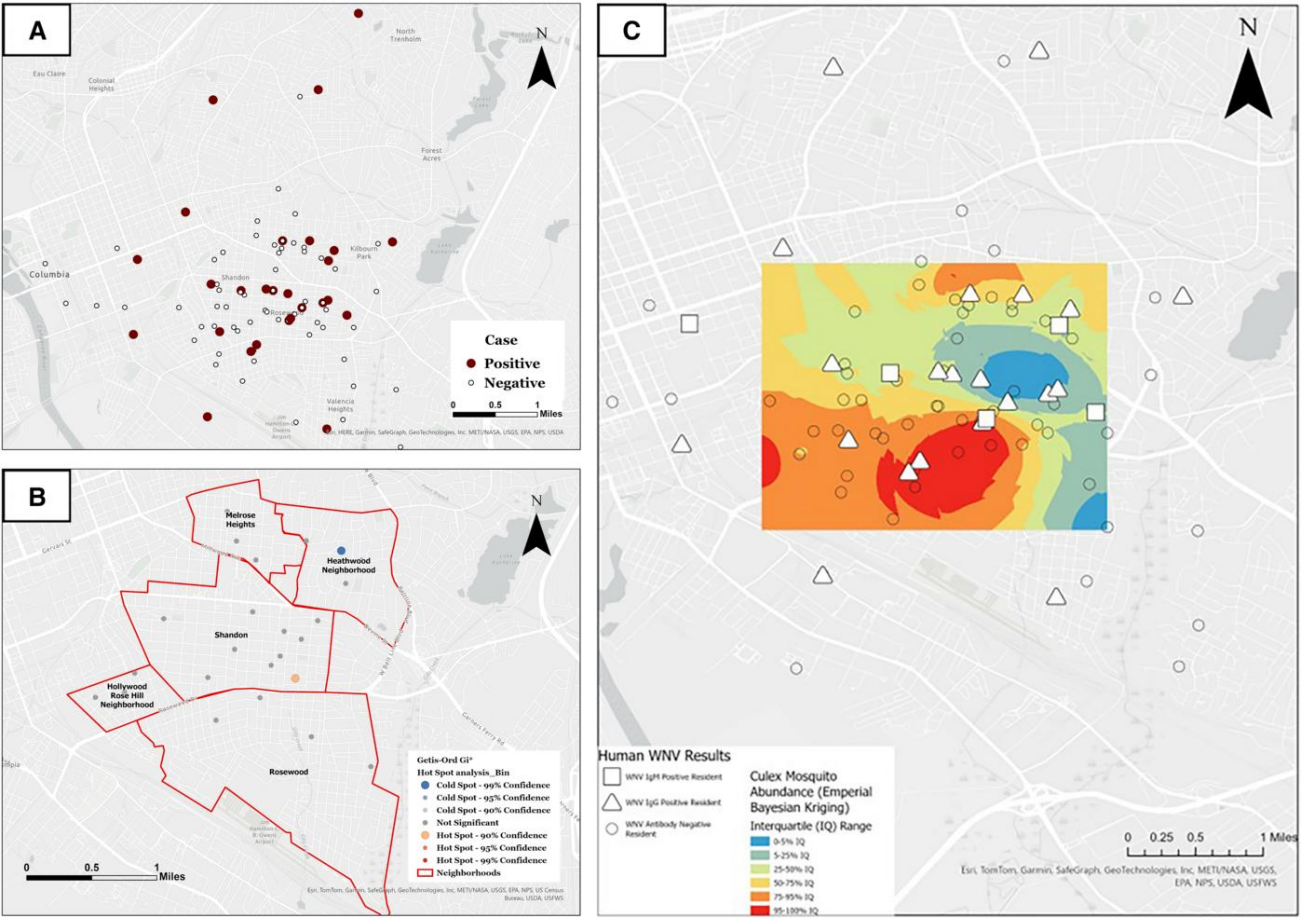
Residents seropositive for West Nile virus (WNV) were widely distributed across five neighborhoods in Richland County, SC. (**A**) Location of serologically positive and negative human cases. (**B**) Spatial clustering results of human WNV cases and seronegative controls. (**C**) Seropositive residential locations overlaid on the *Culex* mosquito abundance distribution map.

A total of 138 residents provided blood samples for the serosurvey (Table 1). West Nile virus IgM seroprevalence was 4.62% (*n* = 6), and the WNV-IgG rate was 17.52% (*n* = 24). No participants had both antibodies simultaneously, resulting in a combined seroprevalence rate of 21.90% (*n* = 30) in Richland County, SC. Compared with the average estimated national WNV seroprevalence of 2.2% and estimated South Carolina seroprevalence of 0.11%, the combined seroprevalence rate in Richland County was notably higher than expected (29–32). Human seropositive residents were geospatially dispersed with one statistical hotspot and a coldspot noted in the study area (Figure 1A and B). Seropositive residents tended to live in areas where the majority of female *Culex* spp. mosquitoes were collected throughout the study period (Figure 1C).

**Table 1:** Epidemiologic risk factors and associations with West Nile virus IgM, West Nile virus IgG, and combined seroprevalence status

	Entire Cohort, *N* = 137 (%)	WNV IgM-Negative, *n* = 113 (%)	WNV IgM-Positive, *n* = 6 (%)	Multivariate *P*-Value OR (95% CI)	WNV IgG-Negative, *n* = 107 (%)	WNV IgG-Positive, *n* = 24 (%)	Multivariate *P*-Value OR (95% CI)	WNV, Combined Seronegative, *n* = 107 (%)	WNV, Combined Seropositive, *n* = 30 (%)	Multivariate *P*-Value OR (95% CI)
Participant characteristics										
Female	79 (57%)	70 (57%)	4 (67%)	NS	63 (56%)	16 (67%)	NS	59 (55%)	20 (67%)	NS
Participant age (median)	48 (range: 18 to 85)	48 (range: 18 to 85)	64 (range: 21 to 75)	NS	47 (range: 18 to 83)	60 (range: 20 to 85)	NS	45 (range: 18 to 83)	62 (range: 20 to 85)	NS
Race: non-Hispanic White	111 (80%)	99 (80%)	6 (100%)	NS	91 (81%)	20 (83%)	NS	85 (79%)	26 (87%)	NS
Race: non-Hispanic Black	10 (7%)	9 (7%)	0	NS	8 (7%)	2 (8%)	NS	8 (7%)	2 (7%)	NS
Race: Hispanic	6 (4%)	4 (3%)	0	NS	4 (4%)	2 (8%)	NS	4 (4%)	2 (7%)	NS
Race: Asian	5 (4%)	5 (4%)	0	NS	4 (4%)	1 (4%)	NS	4 (4%)	1 (3%)	NS
Number of years lived in Columbia, SC (median)	12 (range: 19 to 49)	12 (range: 19 to 44)	23 (range: 16 to 44)	**0.0141**	12 (range: 18 to 39)	16 (range: 22 to 49)	**0.0180**	11 (range: 18 to 39)	16 (range: 21 to 49)	**0.0414**
Number of years lived at current address (median)	2 (range: 1.67 to 5)	4 (range: 0 to 5)	3 (range: 2 to 5)	NS	2 (range: 2 to 5)	2 (range: 2 to 5)	NS	2 (range: 2 to 5)	2 (range: 2 to 5)	NS
Education level										
High school	7 (5%)	5 (4%)	0	NS	4 (4%)	3 (13%)	NS	4 (4%)	3 (10%)	NS
College, associates	52 (38%)	45 (36%)	3 (50%)	NS	43 (38%)	9 (38%)	NS	40 (37%)	12 (40%)	NS
Graduate degree	71 (51%)	67 (54%)	3 (50%)	NS	59 (52%)	12 (50%)	NS	56 (52%)	15 (50%)	NS
Annual household income level (USD)										
<$30,000	11 (8%)	10 (8%)	0	NS	8 (7%)	3 (13%)	NS	8 (7%)	3 (10%)	NS
$30,000–50,000	7 (5%)	6 (5%)	0	NS	6 (5%)	1 (4%)	NS	6 (6%)	1 (3%)	NS
$50,000–75,000	13 (9%)	11 (9%)	2/4 (50%)	NS	11 (10%)	2 (8%)	NS	9 (8%)	4 (13%)	NS
$75,000–100,000	10 (7%)	10 (8%)	0	NS	9 (8%)	1 (4%)	NS	9 (8%)	1 (3%)	NS
$100,000–150,000	30 (22%)	30 (24%)	0	NS	22 (19%)	8 (33%)	NS	22 (21%)	8 (27%)	NS
>$150,000	36 (26%)	32 (26%)	2/4 (50%)	NS	31 (27%)	5 (21%)	NS	29 (27%)	7 (23%)	NS
Household characteristics										
Property has a manicured lawn	77 (55%)	66 (53%)	4 (67%)	NS	62 (55%)	15 (63%)	NS	48 (45%)	14 (47%)	NS
Property has little vegetative growth	40 (29%)	38 (31%)	1 (17%)	NS	33 (29%)	7 (29%)	NS	30 (28%)	5 (17%)	NS
Property contains water features	13 (9%)	10 (8%)	3 (50%)	**0.008 OR 23.70 (2.24 to 250.21)**	11 (10%)	2 (8%)	NS	8 (7%)	4 (13%)	NS
Property has small containers	28 (20%)	26 (21%)	2 (33%)	NS	22 (19%)	6 (25%)	NS	16 (15%)	5 (17%)	NS
Property has bird-friendly items	47 (34%)	45 (36%)	1 (17%)	NS	38 (34%)	9 (28%)	NS	33 (31%)	5 (17%)	NS
Property has tree canopy/shading	76 (55%)	70 (56%)	4 (67%)	NS	63 (56%)	13 (54%)	NS	52 (49%)	12 (40%)	NS
Property contains areas of wild growth	43 (31%)	39 (31%)	2 (33%)	NS	33 (29%)	10 (42%)	NS	28 (26%)	7 (23%)	NS
Mosquito bite frequency										
Mosquito presence around property	90 (65%)	81 (65%)	4 (67%)	NS	73 (65%)	17 (71%)	NS	58 (54%)	15 (50%)	**NS**
Reported higher frequency of mosquito bites on property	75 (54%)	67 (54%)	3 (50%)	NS	64 (57%)	11 (46%)	**0.041 OR 0.33 (0.11 to 0.95)**	51 (48%)	9 (30%)	**0.035 OR 0.344 (0.128 to 0.925)**
Mosquito presence on property in May	29 (21%)	24 (19%)	2 (33%)	NS	27 (24%)	2 (8%)	NS	24 (22%)	2 (7%)	NS
Mosquito presence on property in June	72 (52%)	63 (51%)	4 (67%)	NS	62 (55%)	10 (42%)	**0.028 OR 0.31 (0.10 to 0.88)**	53 (50%)	11 (37%)	**NS**
Targeted strategies										
Regular management of property	74 (53%)	63 (51%)	5 (83%)	NS	59 (52%)	15 (63%)	NS	47 (44%)	15 (50%)	NS
Usage of insect repellent on property	43 (31%)	38 (31%)	2 (33%)	NS	35 (31%)	8 (33%)	NS	27 (25%)	8 (27%)	NS
Tip and toss any standing water	79 (57%)	72 (58%)	4 (67%)	NS	63 (56%)	16 (67%)	NS	52 (49%)	14 (47%)	NS
Presence of dead birds on property ever	44 (32%)	40 (32%)	2 (33%)	NS	36 (32%)	8 (33%)	NS	30 (28%)	8 (27%)	NS
WNV knowledge										
Has heard of WNV	99 (71%)	88 (71%)	5 (83%)	NS	79 (70%)	20 (83%)	NS	74 (69%)	25 (83%)	NS
Knows the threat of WNV to personal health	66 (47%)	58 (47%)	3 (50%)	NS	51 (45%)	15 (63%)	NS	48 (45%)	18 (60%)	NS
Knows the threat of WNV to their community	87 (63%)	78 (63%)	3 (50%)	NS	68 (60%)	19 (79%)	NS	65 (61%)	22 (73%)	NS
Knows the serious impacts of WNV	99 (71%)	87 (70%)	5 (83%)	NS	79 (70%)	20 (83%)	NS	74 (69%)	25 (83%)	NS
Knows WNV is a mosquito-borne disease	97 (70%)	59 (48%)	2 (33%)	NS	77 (68%)	20 (83%)	NS	49 (46%)	16 (53%)	NS
Knows the role of birds in WNV transmission	85 (61%)	75 (60%)	4 (67%)	NS	69 (61%)	16 (67%)	NS	65 (61%)	20 (67%)	NS
Household WNV exposure										
Someone in the house has been infected with a mosquito-borne virus	4 (3%)	3 (2%)	0	NS	3 (3%)	1 (4%)	NS	3 (3%)	1 (3%)	NS
Know of anyone in Columbia, SC, infected with a mosquito-borne virus	31 (22%)	27 (22%)	1 (17%)	NS	26 (23%)	5 (21%)	NS	21 (20%)	5 (17%)	NS
Household member(s) with headaches within the past few months	25 (18%)	22 (18%)	2 (33%)	NS	21 (19%)	4 (17%)	NS	17 (16%)	5 (17%)	NS
Household member(s) with body aches within the past few months	24 (17%)	20 (16%)	3 (50%)	NS	19 (17%)	5 (21%)	NS	14 (13%)	6 (20%)	NS
Household member(s) with joint pains within the past few months	20 (14%)	17 (14%)	2 (33%)	NS	17 (15%)	3 (13%)	NS	11 (10%)	3 (10%)	NS
Household member(s) with a stiff neck within the past few months	13 (9%)	12 (10%)	0	NS	10 (9%)	3 (13%)	NS	7 (7%)	2 (7%)	NS
Household member(s) with vision loss within the past few months	2 (1%)	1 (1%)	1 (17%)	**0.012 OR 79.00 (2.63 to 2,371.69)**	2 (2%)	0	NS	1 (1%)	1 (3%)	NS
Household member(s) are all healthy	53 (38%)	47 (38%)	1 (17%)	NS	40 (35%)	13 (54%)	NS	34 (32%)	11 (37%)	NS

NS = nonsignificant results on multivariate model (*P* >0.05); OR = odds ratio; WNV = West Nile virus.

Values shown in bold represent statistically significant variables (*P* < 0.05).

Among enrolled participants, the average age was 48 years (range 18–85 years); 57% were female, and most were non-Hispanic White (80%). The cohort was predominantly highly educated (51% had a graduate degree) and middle class (48% reported an annual household income of more than $100,000). The average number of years lived in Columbia, SC, was 27 (range 19–42), and the average number of years lived at the current address was 3.4 (range 1.67–5). Individuals who had lived in the city longer were significantly more likely to test positive for WNV IgM (*P* = 0.014), IgG (*P* = 0.018), or either antibody (*P* = 0.041). Knowledge of WNV or pathogen exposure did not differ significantly across seroprevalence categories. Participants who reported a household member with recent vision loss were 79 times more likely (*P* <0.012; 95% CI: 2.63 to 2,371.69) to be WNV IgM-positive, although these results should be interpreted with caution given the wide CI and the low overall count.

Interestingly, those who reported regular lawn management on their property, such as self-application of insecticides, “tip-and-toss” strategies, or using hired services, were not statistically associated with WNV antibody status. However, participants living on properties with water features were 24 times more likely to be IgM-seropositive (*P* = 0.008; 95% CI: 2.24 to 250.22) compared with those without water features on their properties. Some unusual protective findings regarding personal or domestic mosquito exposure and lower odds of WNV seroprevalence were noted. Individuals who reported a mosquito problem around their property at any time of the year had significantly decreased odds of combined WNV seropositivity (*P* = 0.035; odds ratio [OR] = 0.344; 95% CI: 0.128 to 0.925). Those who reported higher mosquito presence on their properties in June, specifically, had significantly reduced odds of WNV IgG seropositivity (*P* = 0.028; OR = 0.31; 95% CI: 0.11 to 0.88). Lastly, those who reported a higher frequency of mosquito bites were significantly less likely to be WNV IgG-positive (*P* = 0.041; OR = 0.33; 95% CI: 0.11 to 0.95).

### Prospective WNV mosquito surveillance.

**Figure 2.**
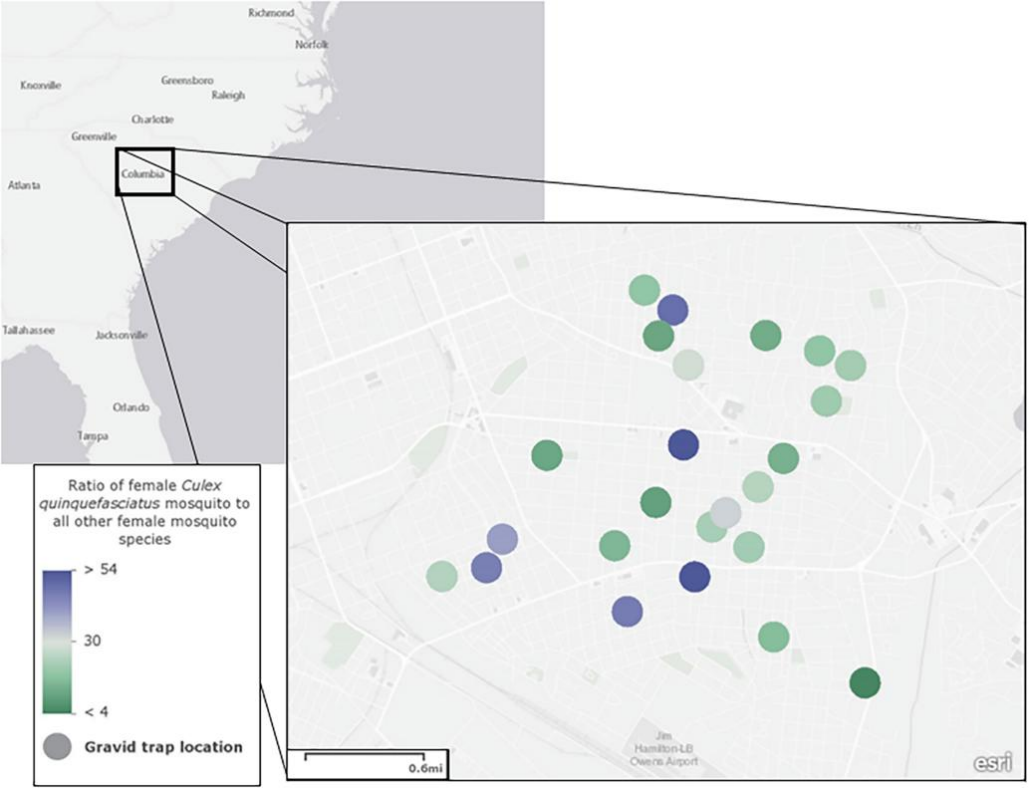
Geospatial variance noted in the ratio of female *Culex quinquefasciatus* versus other female mosquito species in Richland County, SC.

Over 240 trapping nights, a total of 3,687 adult female mosquitoes were collected, comprising 11 different species. *Culex quinquefasciatus* was the most commonly collected adult female mosquito (97% of all collected female mosquitoes), with other medically relevant mosquito species, such as *Anopheles punctipennis* and *Aedes albopictus*, additionally collected. Approximately 2.7% of tested adult mosquito pools were WNV-positive (2 of 73 pools), with 100% homology to the WNV-02 clade (GenBank Accession Number KX547492; Supplemental Figure 2). An additional 673 egg-reared female *Cx. quinquefasciatus* adults were collected during the same time period. Niche partitioning in this species’ breeding habitats was noted, compared with that of other collected species. Specifically, within the same residential block, the ratio of female *Cx. quinquefasciatus* to other female mosquitoes ranged from 4:1 to 54:1 (Figure 2). Data presented in Figure 2 include the total number of female *Cx. quinquefasciatus* in relation to all other species collected at that individual trap site over 10 trap nights.

**Figure 3.**
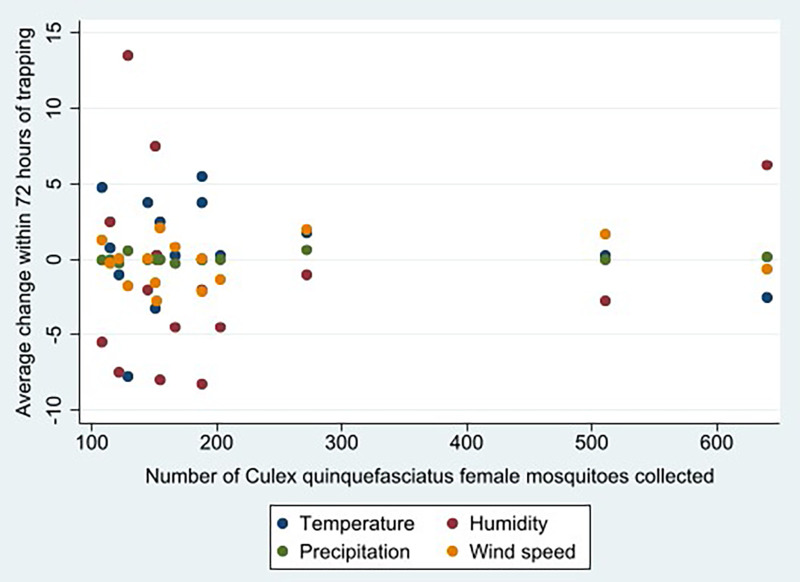
Less climate variation in the 3 days before collection was associated with greater female *Culex quinquefasciatus* abundance.

Greater climate variation in the 72 hours before collection was significantly associated (*P* <0.016) with a lower *Cx. quinquefasciatus* count. As noted in Figure 3, greater variation (+/– 15Δ) in the average temperature, precipitation, humidity, and wind speed was correlated with collecting fewer than 200 female *Cx. quinquefasciatus* during a given trapping event. Interestingly, lower temperatures were associated with higher numbers of female *Cx. quinquefasciatus*, with a bimodal distribution noted (Supplemental Figure 3). Namely, two-thirds of the female *Cx. quinquefasciatus* adults (*n* = 2,585 of 3,919) were collected on days when the temperature was lower than 85°F. Lastly, no land use land classification variables were statistically associated with *Cx. quinquefasciatus* abundance; however, several trends were noted (Supplemental Figure 4). Areas classified as middle-developed-use land, greater tree canopy coverage, lower surface temperatures, and less standing water (normalized difference water index) were correlated with higher *Cx. quinquefasciatus* counts.

## DISCUSSION

The present collective vector–human–pathogen analysis revealed a WNV focal hotspot in central South Carolina. Nearly one-quarter of sampled residents had WNV antibodies, with 5% presenting with IgM antibodies, suggesting recent infection. Traditional epidemiologic risk factors were not associated with seropositive status, yet *Cx. quinquefasciatus* female mosquitoes were found across the geographic study area, and residents had domestic properties supportive of avian populations and mosquito breeding habitats. Approximately 3% of tested adult mosquito pools were WNV-positive, and viral genetic analysis aligned with the historical WN02 clade. A surprising finding was a bimodal distribution of mosquito abundance, with more collected earlier in the season and a notable drop-off in August, which is the time period traditionally associated with peak mosquito transmission activity. Although South Carolina is classified as a low-endemic WNV state (estimated cumulative incidence of <1%),^11^^,^^29^ the study finding of 22% seropositivity highlights the need for prompt public health intervention. Future research is warranted to understand the underlying causes of local hyperendemicity and to identify additional potential transmission hotspots across the continental USA.

The present study is not the first in which WNV “hot spots” have been identified upon further investigation in counties considered by the US Centers for Disease Control and Prevention to have low transmission.^12^^,^^30^ Several studies from Chicago, IL, published over the past 20 years have revealed consistent high-risk foci in landscape-mature, affluent suburban neighborhoods.^31^^–^^33^ Similarly, WNV-seropositive birds were significantly more abundant in suburban neighborhoods compared with downtown, urban sites in Atlanta, GA.^34^ Alternatively, a WNV “hot spot” was noted in lower-income Los Angeles, CA, suburban neighborhoods, although these neighborhoods recently experienced an increase in foreclosed homes and neglected pools, which may suggest that these neighborhoods were recently middle class neighborhoods that experienced an unusual rapid economic decline.^35^ Interestingly, one study revealed that WNV isolates from “hot-spots” exhibited evolutionary rates 10 times greater than the average, suggesting that microenvironments and microclimates play an important role in maintaining hyper transmission.^32^ As these studies collectively reveal, focal transmission likely occurs in areas classified as low-incidence regions. More research is needed to identify these persistent hyperendemic neighborhoods across the United States because public health funding and vector control responses are largely based on diagnosed cases, yet WNV diagnostic orders are known to be underutilized among American medical providers.^16^^,^^36^

The impact of climate change on arboviral diseases threatens geographic expansion and alterations in mosquito behavior, potentially creating more WNV transmission foci nationally. Contemporary models have revealed that *Culex* spp. exhibit unique ecological responses to climate change, with *Culex tarsalis, Culex pipiens*, and *Culex salinarius* being the most likely to expand geographically across the Americas in the foreseeable future.^37^ The current study revealed a bimodal *Cx. quinquefasciatus* adult female abundance curve, which differs from historical findings in which mosquito abundance followed a normal distribution with an August peak.^38^ Laboratory studies have revealed that juvenile and adult *Culex* spp. developmental time and survival decrease at elevated temperatures.^39^^,^^40^ The recent rise in August temperatures has led to higher temperatures than those of *Culex* spp. physiological thermal thresholds,^41^^–^^43^ which is the likely cause of the bimodal *Culex* spp. mosquito abundance patterns noted in the present study. Additionally, this study revealed that lower climate variation was associated with higher mosquito collections. Models have suggested that microclimate thermal heterogeneity is common in natural environments,^44^^,^^45^ which could benefit public health if urban environments begin to experience more sudden climate changes in summer months, leading to fewer favorable *Culex* mosquito breeding environments.

Some unusual epidemiologic risk factors for human WNV seropositivity were identified in the present study. Individuals who reported a higher frequency of mosquitoes around their domestic property, especially in May or June, or more mosquito bites while on their property, were statistically less likely to test positive for WNV antibodies. Although none of the targeted mosquito abatement strategies were statistically significant, individuals with known domestic mosquito problems may have been more likely to take personal preventive measures, thereby reducing the risk of mosquito-borne disease transmission. Similarly, some established WNV risk factors were not statistically associated with seroprevalence: having wild, old-growth vegetation or bird-friendly items on the property; educational or socioeconomic status; older age; or the presence of dead birds on the property.^33^^,^^35^^,^^46^^,^^47^ Although it is probable that WNV transmission dynamics have changed since its first introduction 25 years ago, it is also likely that the study sample was not adequately powered to detect social–ecological factors, and future studies are warranted to validate contemporary WNV transmission factors.

The present study had a few limitations worth noting. First, a cross-sectional study design was used to test residents, which limited the ability to confirm active WNV infection throughout the summer season. Additionally, the potential for participant self-selection bias cannot be ruled out. Although recruitment was performed at three socioeconomically diverse locations, White, affluent individuals are historically more likely to participate in research studies, and the study population similarly reflected this.^48^ Second, the present study was geographically focused in an area reported to be WNV-endemic by local state public health department officials, which could lead to an artificially high seroprevalence rate that is not representative of the entire county or region. Furthermore, the present study was conducted over one summer, which is not representative of interannual climate fluctuations or land use and land classification changes. Prospective studies with an expanded geographic reach would be beneficial for further understanding the micro-environmental factors underlying hyperendemic transmission foci.

## CONCLUSION

As WNV clinical attention and public health funding wane, the medical impact of this neuroinvasive arbovirus remains. Twenty-five years after its introduction, WNV continues to circulate in the United States, and the present study reveals that human infections are likely occurring at a higher rate than previously anticipated. The lack of traditional epidemiological risk factors, combined with the 10-fold higher seroprevalence among central South Carolinians, warrants further study to better understand the environmental factors leading to hyperendemicity and the contemporary WNV clinical profile. The elevated IgM seroprevalence rate observed in the present study raises the question of whether WNV is becoming less virulent, leading to more asymptomatic infections, whether clinicians are failing to diagnose veritable cases, or whether viral tissue tropisms have evolved over time, leading to different clinical manifestations and pathognomonic indicators. West Nile virus research has precipitously dropped since 2006, leading to clinical and vector guidelines based on decades-old evidence. As this virus continues to evolve, will clinicians and public health professionals evolve too?

## Supplemental Materials

10.4269/ajtmh.25-0305 Supplemental Materials
