# Supplementary material for: Ecologic Factors Contributing to West Nile Virus Hyperendemicity in Central South Carolina: An Integrated Vector–Human–Environmental Study
Source: Am J Trop Med Hyg. 2026 Feb 5;114(4):742–50. doi: 10.4269/ajtmh.25-0305 (PMC13045586; doi:10.4269/ajtmh.25-0305)

Supplemental Figure 1. Graphical representation of the integrated environmental, mosquito and human study components.

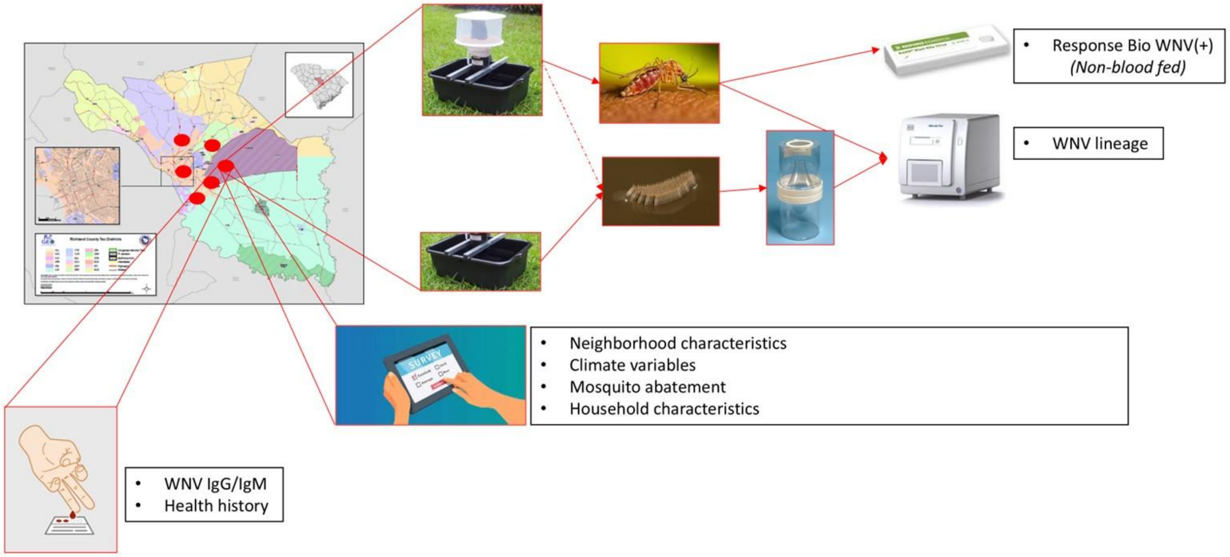

Supplemental Figure 2. West Nile virus detected in Richland County, SC *Culex quinquefasciatus* mosquitos most closely aligned with WN02 clade.

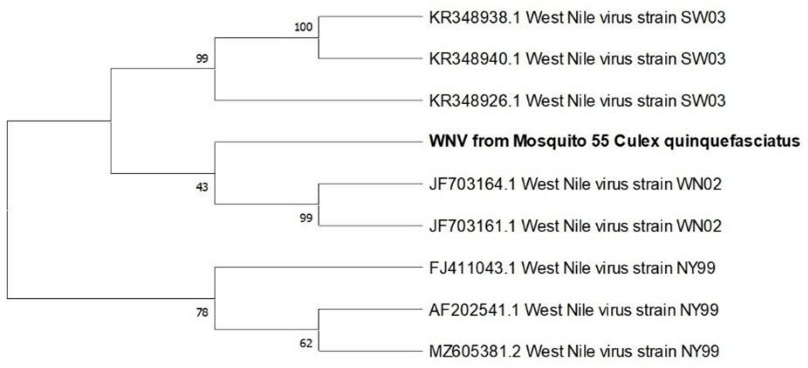

Supplemental Figure 3. Bimodal distribution of female *Culex quinquefasciatus* abundance noted in response to peak seasonal temperature.

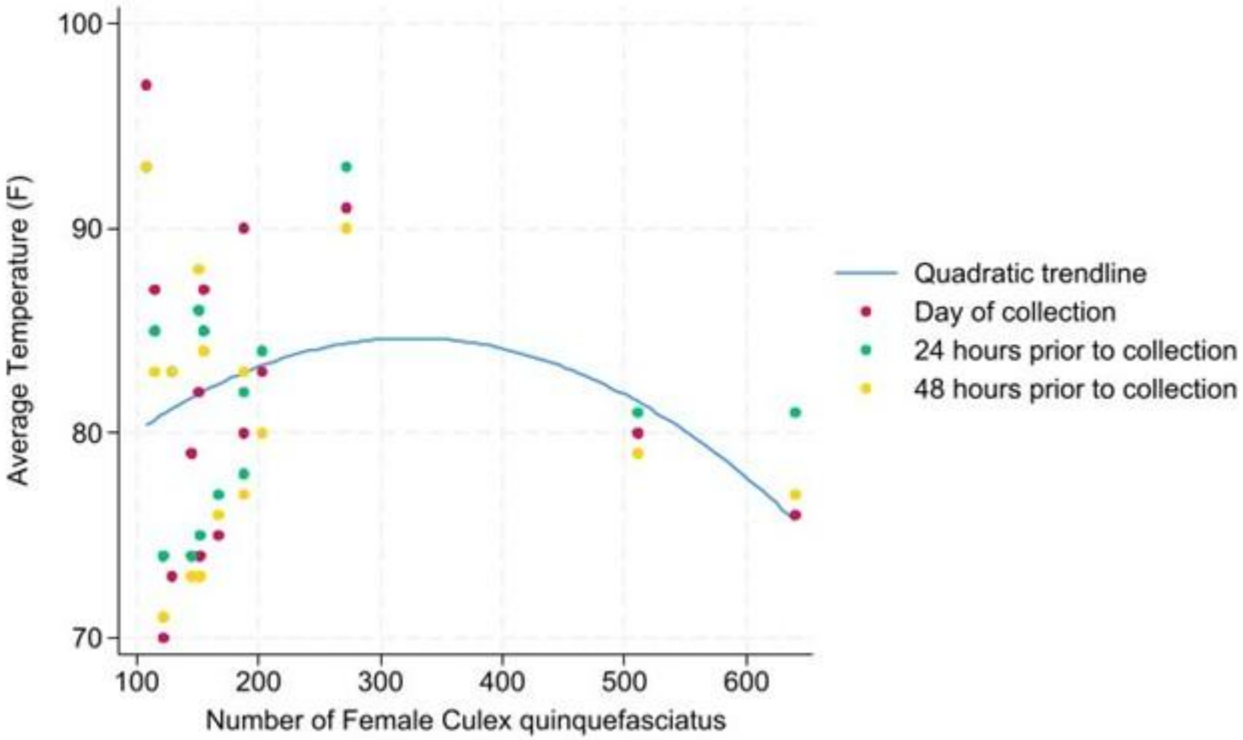

Supplemental Figure 4. Remote sensing variables were not statistically associated with female *Culex quinquefasciatus* abundance, although notable trends were observed**.** (**A**) Developed land use. (**B**) Surface temperature. (**C**) Normalized difference water index. (**D**) Tree canopy.

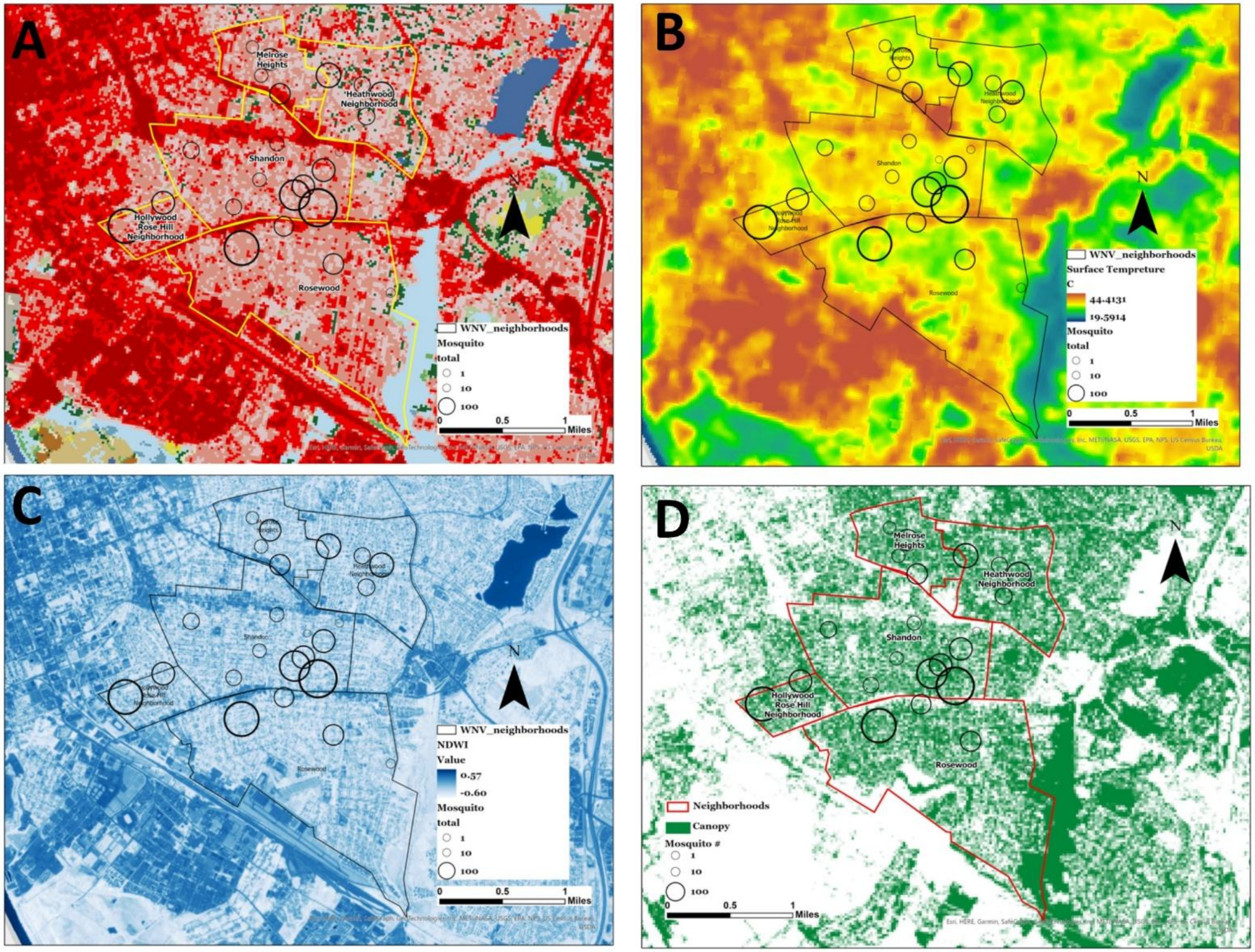

Supplement: Supplemental Materials [file tpmd250305.SD1.pdf]
